# Porcine Blood: An Eco-Efficient Source of Multifunctional Protein Hydrolysates

**DOI:** 10.3390/foods15020254

**Published:** 2026-01-10

**Authors:** Sandra Borges, Joana Odila, Glenise Voss, Rui Martins, André Almeida, Manuela Pintado

**Affiliations:** 1 CBQF—Centro de Biotecnologia e Química Fina—Laboratório Associado, Escola Superior de Biotecnologia, Universidade Católica Portuguesa, Rua Diogo Botelho 1327, 4169-005 Porto, Portugal; 2 SEBOL—Comércio e Indústria do Sebo, S.A., 2660-119 Loures, Portugal; 3 I.T.S.—Indústria Transformadora de Subprodutos S.A., 2100-406 Loures, Portugal

**Keywords:** porcine by-products, blood hydrolysis, bioactive peptides, gastrointestinal digestion, techno-functional properties

## Abstract

Porcine blood is a major slaughterhouse by-product and a sustainable source of high-quality proteins with potential food and nutraceutical applications. This study valorized porcine whole blood (WB, 6.7 ± 0.1% protein) and red cell fraction (CF, 50.4 ± 0.2% protein) through alcalase hydrolysis, generating hydrolysates (WBH and CFH) with bioactive and techno-functional properties. Optimal hydrolysis conditions, defined as enzyme-to-substrate (E/S) and incubation time yielding the highest degree of hydrolysis (DH) with cost-effective enzyme usage, were 1% E/S for 4 h (WBH) and 2.5% E/S for 4 h (CFH). WBH showed a higher DH (59.5 ± 2.6%) than CFH (30.8 ± 3.3%). Antioxidant assays revealed higher ABTS activity in CFH (14.1 vs. 11.1 mg ascorbic acid equivalents/g, *p* < 0.05), while both exhibited similar ORAC values (166.8–180.2 mg Trolox equivalents/g, *p* > 0.05). After simulated gastrointestinal digestion, ABTS activity was preserved, whereas ORAC decreased (~40%). ACE inhibitory activity was also pronounced, particularly in CFH (IC_50_ = 59.5 µg protein/mL), but digestion converged values between hydrolysates (118–135 µg protein/mL). Techno-functional tests showed moderate emulsifying activity (~40%), with CFH displaying markedly higher oil absorption (4.79 vs. 1.31 g oil/g). Considering the limited information on porcine blood hydrolysates under gastrointestinal conditions, these findings provide new insights into their stability and support their potential as multifunctional ingredients for health-promoting foods and functional formulations.

## 1. Introduction

The global demand for animal-based protein has increased, raising significant concerns regarding environmental impact and food security [1]. Blood is a by-product of slaughterhouses in the meat industry, at large volumes. Porcine blood accounts for around 7% of total body weight and is composed of 15–17% high-quality protein, including albumin, globulin and hemoglobin, making it a readily available and cost-effective protein source [2,3]. The volume of pig blood produced annually, estimated at approximately 39,216 tons, has a substantial impact on the environment if not properly managed [2]. Livestock blood waste is recognized as an environmentally critical effluent due to its exceptionally high organic load and nutrient concentration, which translate into elevated biochemical and chemical oxygen demand. When discharged without adequate treatment, blood waste can lead to severe water and soil pollution, oxygen depletion in aquatic ecosystems and the emission of greenhouse gases during decomposition, representing a significant environmental burden [4]. The principal consumer markets for animal blood-derived food products, such as blood puddings, sausages, cookies, cakes and breads, are concentrated in Europe and Asia, where these items are traditionally integrated into local diets. In contrast, the remaining fractions of collected blood are typically utilized in low-value applications, including animal feed and agricultural fertilizers, or are discarded as liquid waste, resulting in substantial loss of potentially valuable biological resources [5,6].

Blood is a slightly alkaline, heterogeneous fluid composed of cellular components, including red blood cells (erythrocytes), white blood cells (leukocytes) and platelets suspended in plasma. Plasma accounts for about 65–70% of the total blood volume, while the remaining 30–35% consists of these cellular elements [7,8]. There is growing interest in innovative technologies and efficient processing strategies that enable the full utilization of these proteins by converting them into high-value products. Enzymatic hydrolysis enables the production of protein hydrolysates enriched with bioactive peptides that possess antioxidant and angiotensin-converting enzyme (ACE) inhibitory activities, presenting promising potential for managing oxidative stress and hypertension [9,10]. This is particularly relevant given that hypertension affects approximately 1.28 billion adults worldwide [11]. Previous research has highlighted porcine blood proteins as a particularly effective source of such bioactive compounds [3,12,13].

Proteins extracted from animal blood have potential applications as food additives in human nutrition. Understanding their functional properties is essential for optimizing their incorporation into existing food products or for the development of novel formulations. Emulsification is essential in the production of restructured products like sausages and mortadella [14]. Oil absorption capacity is another key attribute, as it influences mouthfeel and flavor retention [15].

The efficacy of bioactive protein hydrolysates as functional food ingredients or nutraceuticals largely depends on their stability and bioavailability under physiological conditions. Simulated gastrointestinal digestion models are commonly employed to assess the behavior and integrity of bioactive peptides during digestion [16]. However, limited data exist on the bioavailability of protein hydrolysates derived from porcine blood [17].

Although several studies have investigated the production of bioactive peptides from animal blood, important knowledge gaps remain. Most available research focuses on isolated protein fractions or uses anticoagulated blood, and comparative analyses of WB and its CF under enzymatic hydrolysis are scarce. Additionally, limited information exists regarding the combined assessment of bioactive, techno-functional and digestive stability properties of porcine blood hydrolysates. These gaps restrict the full valorization of this by-product in food applications.

This study hypothesizes the possibility of generating bioactive protein hydrolysates with antioxidant and ACE inhibitory activities from both the raw and cellular fractions of porcine blood, without the use of anticoagulants, enhancing their applicability in functional food systems. A secondary hypothesis is that these bioactive peptides will retain their biological activities following simulated digestion, indicating potential for absorption, stability and efficacy at the target site.

The broader goal of this work is to promote the valorization of porcine blood, a major slaughterhouse by-product, by developing high-value bioactive ingredients. Such applications may contribute to improved health outcomes, better food preservation and enhanced textural properties in food formulations.

## 2. Materials and Methods

### 2.1. Materials

Porcine blood was sourced from ETSA (Loures, Portugal), a company specialized in the collection and processing of animal by-products. Two types of substrates were used in this study: (i) whole blood (WB) containing 6.7 ± 0.1% protein, which was stored immediately at 4 °C, and (ii) red cell fraction (CF) containing 50.4 ± 0.2% protein, obtained by direct steam-induced coagulation, performed by injecting steam (3 bar, 133 °C) into fresh blood for 2–5 s, enough to ensure complete protein coagulation, in a continuous process. This is followed by decantation and removal of the supernatant plasma.

These two fractions were selected as substrates because they represent the main industrially available forms of porcine blood and exhibit distinct protein compositions, offering materials with different technological and biological relevance for enzymatic hydrolysis.

All reagents were purchased from Sigma-Aldrich (St. Louis, MO, USA) unless mentioned otherwise.

### 2.2. Enzymatic Hydrolysis of Porcine Blood

Porcine blood samples (WB and CF) were hydrolyzed using alcalase (2600 U/mL). For all hydrolysis assays, 25 g of raw material were suspended in 75 mL of 0.1 M potassium phosphate buffer (pH 8.0), and different amounts of alcalase were added according to the E/S ratios tested. In this optimization study, the enzyme loading (E/S), expressed as the percentage of enzyme solution added (*v*/*w*) relative to the mass of raw material, and the hydrolysis time were varied. The impact of these conditions was assessed by monitoring the DH and evaluating the molecular weight distribution of the resulting peptides. For the purpose of this study, the effectiveness of the hydrolytic process was defined as the combination of enzyme loading and hydrolysis time that resulted in a high DH together with a clear shift toward low-molecular-weight peptides. Hydrolysis was conducted under standard operational conditions recommended for alcalase activity, i.e., at 50 °C and pH 8.0 [10]. Aliquots were collected at specific timepoints: 0, 2, 4, 6, 8, and 24 h. To stop enzymatic activity, samples were heat-treated at 95 °C for 15 min. The hydrolysates were then centrifuged at 8000 rpm for 10 min at 4 °C, and the supernatants were collected and stored at −80 °C for further analysis.

All hydrolysis reactions were carried out in duplicate, with control samples prepared under the same conditions but without enzyme addition.

#### 2.2.1. Determination of Degree of Hydrolysis

The DH was determined by quantifying free amino groups using the TNBS (2,4,6-trinitrobenzenesulfonic acid) method, as described by Borges et al. [18]. Briefly, 50 µL of each sample was mixed with 125 µL of 200 mM sodium phosphate buffer (pH 8.2) and 50 µL of 0.025% TNBS solution. The reaction was incubated at 45 °C for 1 h, and absorbance was measured at 340 nm. A standard curve was prepared using L-leucine (0.078–2.5 mM).

To express the results as DH, the amount of free amino groups released during hydrolysis was related to the total number of peptide bonds in the substrate. The maximum amino group content (Lmax) was determined by complete acid hydrolysis of the raw material with 6 M HCl at 105 °C for 24 h. The hydrolyzed mixture was then filtered, and the supernatant neutralized with 6 M NaOH prior to TNBS quantification. DH was calculated according to the following equation:

DH (%) = [(Lt − L0)/(Lmax − L0)] × 100

where Lt is the concentration of free amino groups at hydrolysis time t, L0 is the amino group content of the untreated substrate, and Lmax corresponds to the amino groups released after complete acid hydrolysis. An independent estimation of total peptide bonds based on Kjeldahl protein content of the raw materials confirmed the plausibility of the Lmax values obtained by TNBS. All measurements were performed in duplicate and expressed in % of hydrolysis.

#### 2.2.2. Molecular Weight Distribution Analysis

The molecular weight (MW) distribution of porcine blood hydrolysates (WBH and CFH) was assessed by gel filtration chromatography using a fast protein liquid chromatography (FPLC) system (ÄKTA Pure, GE Healthcare Life Sciences, Chicago, IL, USA). Two gel filtration columns were employed in series: Superdex 200 Increase 10/300 GL and Superdex Peptide 10/300 GL. The mobile phase consisted of 0.025 M phosphate buffer (pH 7.0), supplemented with 0.2 g/L sodium azide and 8% NaCl, operated at a flow rate of 0.5 mL/min. Elution was monitored at 280 nm. A standard MW calibration curve was generated using the following proteins and peptides: thyroglobulin (669 kDa), ferritin (440 kDa), aldolase (158 kDa), conalbumin (75 kDa), ovalbumin (44 kDa), carbonic anhydrase (29 kDa), ribonuclease A (14 kDa), and whey peptide (1 kDa). Results were expressed as milli-absorbance units (mAU) per eluted volume (mL) [19].

### 2.3. Characterization of Scaled-Up Porcine Blood Hydrolysates

The scale-up to a 5 L reactor was performed to demonstrate the practical feasibility of applying the optimized hydrolysis conditions beyond bench scale and to generate sufficient amounts of hydrolysates for subsequent bioactive and techno-functional characterization. Following the enzymatic hydrolysis kinetics study of porcine blood samples, optimal conditions were selected for the scale-up production of hydrolysates from whole blood (WBH) and the red cell fraction (CFH). For the scale-up trials, the substrate-to-reaction medium proportion established during the optimization phase (1:3, *w*/*v*) was maintained, adjusting the mixture to a final volume of 5 L. The enzymatic loadings identified as optimal at bench scale, 1% (*v*/*w*) for WB and 2.5% (*v*/*w*) for CF with a reaction time of 4 h, were applied directly to the larger volume, while all operational variables (temperature and pH) were kept identical to those used in the kinetic assays to ensure process consistency. After enzymatic hydrolysis, the resulting supernatants, rich in protein hydrolysates, were dried using a freeze dryer (Armfield SB4 model, Hampshire, UK) and stored in a desiccator until further characterization.

#### Determination of Total Protein and Free Amino Acid Content

Total protein content was quantified using the Kjeldahl method, applying a nitrogen-to-protein conversion factor of 6.25. Free amino acid content in the porcine blood hydrolysates (WBH and CFH) was analyzed through pre-column derivatization with orthophthalaldehyde (OPA). In this method, isoindole-type fluorescent derivatives were formed in an alkaline borate buffer (pH 10.4) by reaction of OPA, 2-sulfanylethanol and the primary amine groups of the amino acids. The resulting derivatives were separated using reverse-phase high-performance liquid chromatography (RP-HPLC) (Beckman Coulter, Indianapolis, IN, USA) equipped with a fluorescence detector (Waters, Milford, MA, USA), following the procedure described by Proestos et al. [20]. For each analysis, 100 µL of the sample was derivatized and 20 µL of the resulting solution was injected. All analyses were carried out in duplicate, and quantification was performed using calibration curves prepared with pure amino acid standards. Results were expressed as mg/g of protein.

### 2.4. Bioactive Properties of Porcine Blood Hydrolysates

#### 2.4.1. Analysis of Antioxidant Activity

##### 2,2-Azino-bis-3-Ethylbenzothiazoline-6-sulphonic Acid (ABTS) Scavenging Assay

The free radical-scavenging capacity of WBH and CFH was assessed using the ABTS radical decolorization assay, as described by Re et al. [21]. The ABTS•^+^ radical cation was generated by reacting ABTS with potassium persulfate. Subsequently, 1 mL of the ABTS solution was mixed with the sample and incubated for 6 min. The absorbance was then measured at 734 nm. A calibration curve was constructed using ascorbic acid within the concentration range of 0.063 to 0.250 mg/mL. All measurements were carried out in triplicate, and the results were expressed as mg of ascorbic acid equivalents/g of dry extract (mg AAE/g).

##### Oxygen Radical Absorbance Capacity (ORAC) Assay

The ORAC assay was conducted according to the procedure described by Ou et al. [22], with minor modifications. WBH and CFH samples were dissolved in 75 mM phosphate buffer (pH 7.4), and 120 μL of fluorescein solution (70 nM) was added to each well of a black 96-well microplate (Nunc, Roskilde, Denmark). The plate was incubated at 40 °C for 10 min before initiating the reaction by adding 60 μL of 2,2′-azobis(2-amidinopropane) dihydrochloride (AAPH, 14 mM). Fluorescence was monitored every minute for 140 min using a microplate reader (Synergy H1, Agilent Technologies, Inc. (BioTek Instruments), Santa Clara, CA, USA) with excitation and emission wavelengths set at 485 nm and 528 nm, respectively. The area under the fluorescence decay curve (AUC) was calculated for each sample by integrating the relative fluorescence over time. Trolox, at concentrations ranging from 9.98 × 10^−4^ to 7.99 × 10^−3^ μmol/mL, was used as a standard. Results were expressed as mg of Trolox equivalents/gr of dry extract (mg TE/g).

#### 2.4.2. Measurement of Angiotensin-Converting Enzyme (ACE) Inhibitory Effect

The ACE inhibitory activity of WBH and CFH was evaluated following the method described by Borges et al. [18]. The synthetic substrate *o*-Abz-Gly-*p*-Phe(NO_2_)-Pro-OH (0.45 mM) (Bachem, Bubendorf, Switzerland) was used, and the assay was performed in the presence of ACE (peptidyl-dipeptidase A, EC 3.4.15.1) at a final activity of 0.04 U/mL, in a buffer solution at pH 8.3 containing 0.1 mM ZnCl_2_. The reaction mixtures were incubated at 37 °C for 45 min, and fluorescence was measured using a microplate reader, with excitation and emission wavelengths set at 350 nm and 420 nm, respectively. All measurements were performed in duplicate. The IC_50_ values were determined by non-linear regression analysis and expressed as µg protein/mL.

### 2.5. Simulation of Gastrointestinal Tract Conditions

WBH and CFH samples were subjected to simulated gastrointestinal digestion according to the protocol described by Amorim et al. [23]. The oral phase was initiated by adding 0.6 mL of α-amylase solution (100 U/mL), followed by incubation at 37 °C for 1 min under agitation (200 rpm). For the gastric phase, the pH was adjusted to 2.0 using concentrated HCl (6 M), and the samples were incubated with pepsin (25 mg/mL; from porcine stomach mucosa, pepsin A, 250 U/mg) at a ratio of 0.05 mL per mL of sample for 60 min at 37 °C and 130 rpm. In the intestinal phase, the pH was adjusted to 6.0 using NaHCO_3_ (1 M), followed by the addition of pancreatin (2 g/L; from porcine pancreas, 8× USP) and bile salts (12 g/L) at a volume ratio of 0.25 mL per mL of sample. The mixture was incubated for 120 min at 37 °C and 45 rpm. All digestions were carried out in duplicate. Following the digestion process, antioxidant activity and ACE inhibitory capacity were evaluated using the methodologies previously described.

### 2.6. Techno-Functional Properties

#### 2.6.1. Emulsifying Property and Stability

The emulsifying capacity and emulsion stability of WBH and CFH samples were assessed following the methodology described by Chaparro et al. [24], with minor modifications. Samples were dispersed in distilled water at a concentration of 10 mg/mL (this concentration corresponded to 9.03 mg protein/mL and 8.55 mg protein/mL for WBH and CFH, respectively) and then homogenized with sunflower oil in a 1:1 (*v*/*v*) ratio. The resulting emulsions were centrifuged at 1100 rpm for 5 min, and the height of the emulsified layer and the total volume in the tube were measured. Emulsifying capacity was expressed as a percentage (%). To evaluate emulsifying stability, the emulsions were heated at 80 °C for 30 min prior to centrifugation under the same conditions (1100 rpm, 5 min). The height of the emulsified layer after heating and the total volume before heating were recorded. Emulsifying stability was also expressed as a percentage (%).

#### 2.6.2. Oil Absorption Capacity

The oil absorption capacity of WBH and CFH was determined following the method described by Borges et al. [18]. Each sample was mixed with sunflower oil at a ratio of 1:10 (*w*/*v*) and incubated in a water bath at 60 °C for 30 min. After incubation, the mixtures were centrifuged at 1000 rpm for 15 min. The supernatant was carefully decanted, and the retained oil was weighed. The oil absorption capacity was calculated and expressed as the amount of oil retained per gram of sample (g oil/g sample).

### 2.7. Statistical Analysis

The IBM^®^ SPSS^®^ Statistics 26 program was utilized for statistical analysis. Data normality was evaluated using the Shapiro–Wilk test. Differences among more than two groups were assessed using one-way ANOVA for normally distributed data, followed by Tukey’s HSD post hoc test, or the Kruskal–Wallis test for non-normally distributed data. Comparisons between two groups were performed using Student’s t-test for normal distributions and the Mann–Whitney test for non-normal distributions. A significance level of 0.05 was adopted for all analyses.

## 3. Results and Discussion

### 3.1. Enzymatic Hydrolysis of Porcine Blood

**Figure 1:**
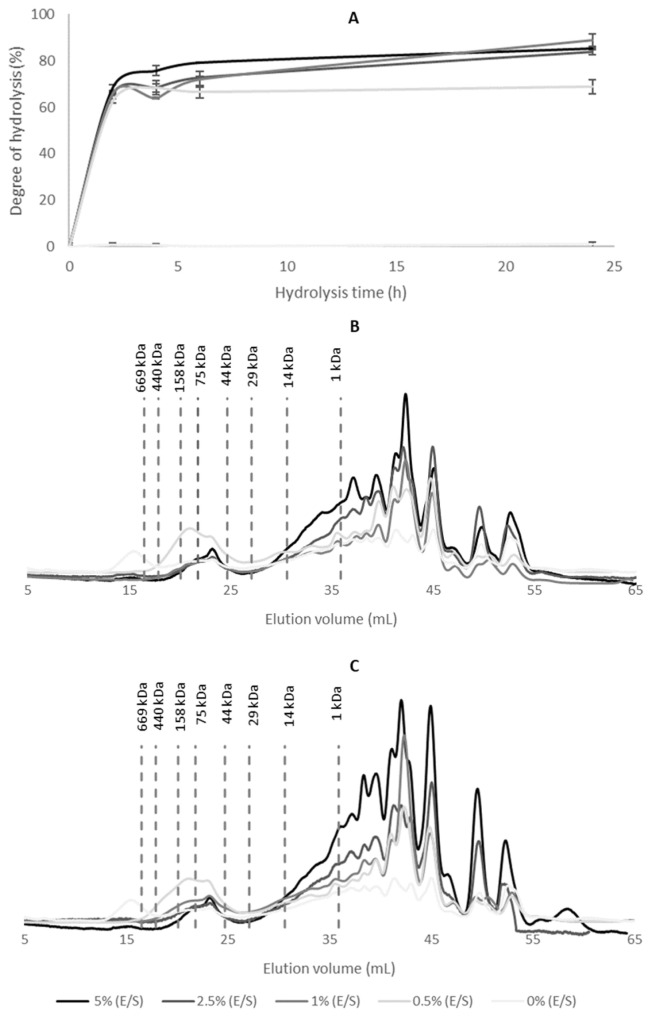
Enzymatic hydrolysis kinetics of WB using alcalase at different E/S ratios: (**A**) DH monitored over 24 h; (**B**) MW distribution after 2 h of hydrolysis; (**C**) MW distribution after 4 h of hydrolysis. Control samples without enzyme (0% E/S) were included. MW markers are indicated.

The enzymatic hydrolysis kinetics of WB using the commercial enzyme alcalase at E/S ratios of 5%, 2.5%, 1%, and 0.5% were assessed by monitoring the DH over time (Figure 1A). The DH, defined as the number of peptide bonds cleaved during the hydrolysis process, is a key parameter for assessing the extent of enzymatic hydrolysis [17]. In the context of this study, the effectiveness of the hydrolytic process was interpreted based on the extent of DH and the evolution of peptide molecular weight profiles, allowing the identification of the hydrolysis conditions that promoted both greater peptide bond cleavage and the formation of lower MW fractions.

After 2 h of hydrolysis, the DH values were relatively consistent across all E/S ratios, ranging from 62.5% to 68.5%. These relatively high DH values can be attributed to the high solubility and structural accessibility of plasma proteins present in WB, which are rapidly hydrolyzed by alcalase. In addition, the TNBS method used in this study typically yields higher numerical DH values compared with methods such as OPA or pH-stat, as it quantifies all free amino groups released during hydrolysis [25]. Similar behavior has been reported for whey protein hydrolysates, where highly soluble proteins also reach DH values above 50% when assessed by TNBS [26].

Overall, the hydrolysis kinetics indicated that most peptide bond cleavage occurred within the first 2 h, followed by a clear tendency toward a stationary phase. This behavior is consistent with previous studies using alcalase, where rapid peptide bond cleavage occurs at early reaction stages, particularly for soluble protein substrates. Although additional sampling within the first 2 h could provide finer kinetic resolution, the DH values obtained at 2 h were already representative of near-maximal hydrolysis and sufficient for the selection of optimal enzyme loading and reaction time. However, by 4 h, a further increase in DH was observed only at the highest E/S ratio of 5%. This trend is characteristic of enzymatic hydrolysis, which typically involves a rapid initial cleavage of peptide bonds, followed by a plateau [18,27]. Several studies suggested that alcalase is most effective during the early stages of the reaction. Bao et al. [28] demonstrated that hydrolysis of egg yolk using alcalase at a 1.2% E/S ratio reached near-maximal DH within the first 60 min, with no significant increase observed after 150 min, indicating that most peptide bond cleavage occurred within the initial 30 min. A similar pattern was reported for yellowfin tuna stomachs hydrolyzed with Alcalase 2.4 L across E/S ratios from 0.2% to 3%, where DH increased rapidly during the first 90 min [29]. This stationary phase in DH may be attributed to several factors, including the limited availability of cleavable peptide bonds, competition between accumulated peptides and remaining substrate, decreased enzyme activity due to pH fluctuations or partial enzyme autolysis [30]. Such secondary or slower hydrolytic phases have also been reported in other protein substrates, where peptide rearrangements or continued cleavage of previously inaccessible regions can occur beyond the initial rapid stage of hydrolysis [19].

To evaluate the changes in MW distribution during hydrolysis, peptide profiles were analyzed for hydrolysates obtained at 2 and 4 h (Figure 1B and Figure 1C, respectively). The control sample (0% E/S) exhibited a dominant chromatographic peak at an elution volume of approximately 16 mL, corresponding to intact high-MW proteins (~600 kDa), which is consistent with the presence of plasma proteins such as immunoglobulin M (~970 kDa) or multimeric glycoproteins like von Willebrand factor (>500 kDa) [31,32], indicating the presence of unhydrolyzed native proteins. This peak was absent in all alcalase-treated samples, confirming effective proteolytic activity. However, the sample hydrolyzed with 0.5% E/S displayed a pronounced peak between 18–25 mL, corresponding to proteins in the 40–350 kDa range, suggesting incomplete hydrolysis at this enzyme concentration. In contrast, treatment with 1% E/S resulted in substantial protein degradation, with the formation of low-MW peptides (<14 kDa), particularly evident after 4 h of hydrolysis (Figure 1C). Considering that porcine WB contains approximately 6.7% protein, even moderate enzyme concentrations are sufficient to achieve substantial hydrolysis. Based on the balance between hydrolytic efficiency and cost-effectiveness, the condition of 1% E/S ratio for 4 h was selected for scaling up the production of WBH. This condition offered an optimal compromise between enzymatic performance and economic viability.

**Figure 2:**
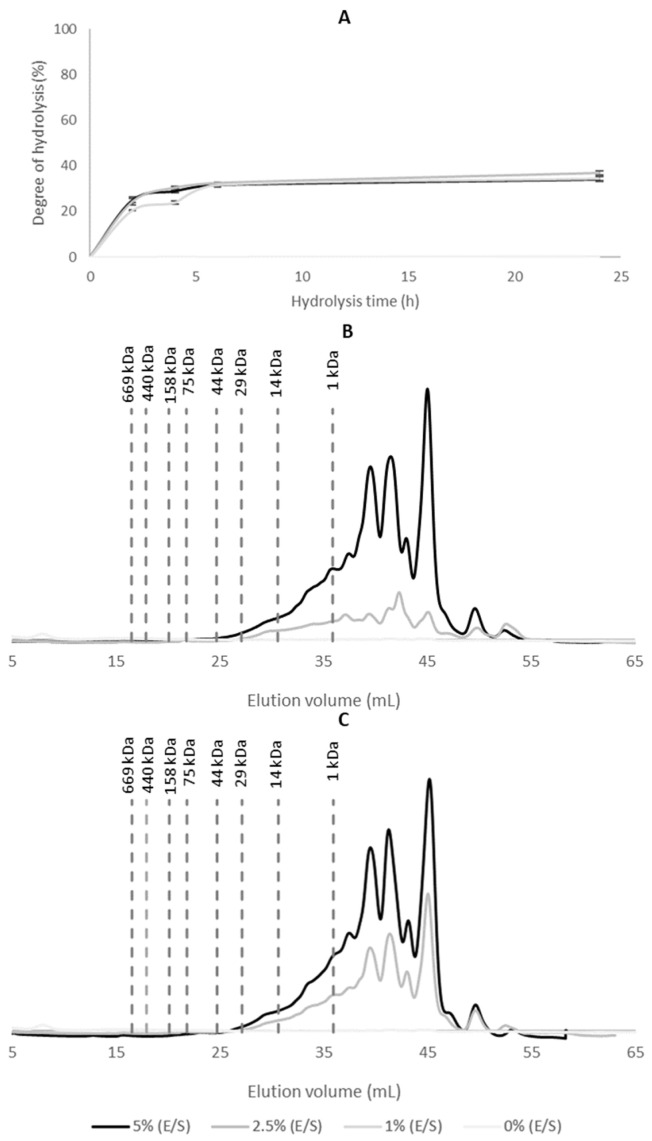
Enzymatic hydrolysis kinetics of CF using alcalase at different E/S ratios: (**A**) DH monitored over 24 h; (**B**) MW distribution after 2 h of hydrolysis; (**C**) MW distribution after 4 h of hydrolysis. Control samples without enzyme (0% E/S) were included. MW markers are indicated.

The DH was also monitored during the enzymatic treatment of the CF with alcalase to evaluate its hydrolytic potential (Figure 2A). As observed for WB hydrolysis, the enzymatic activity was most pronounced within the first 2 h, followed by a slower progression toward a stationary phase.

When using enzyme concentrations of 5% and 2.5% (E/S), DH values remained relatively stable over time, reaching approximately 25% at 2 h and 30% at 4 h, indicating limited hydrolysis after the initial phase. Similar DH values were reported by Pérez-Gálvez et al. [33], that hydrolyzed porcine blood meal with Alcalase^®^ 2.4 L under conditions of 50–60 °C, pH 6–7.5, and E/S ratios of 5–10%, achieving a maximum DH of 28.89%. Additionally, Alves et al. [34] achieved a DH of 22.7% when hydrolyzing chicken blood meal with Alcalase^®^ 2.4 L at 60 °C, pH 8.0, and a 5% E/S ratio for 90 min. These results further support the efficiency of alcalase in hydrolyzing animal blood-derived substrates under moderate enzymatic conditions.

Given the similarity in DH values at time points of 2 h and 4 h, the peptide profiles of the resulting hydrolysates were further investigated by FPLC (Figure 2B,C). FPLC analysis confirmed the formation of low MW peptides in the hydrolyzed samples compared to the control. The control sample (0% E/S) exhibited no significant chromatographic peaks, indicating the limited solubility of CF proteins in their native aggregated form. In contrast, enzymatic hydrolysis promoted the solubilization of the CF fraction, leading to the appearance of multiple peaks in the chromatograms. The majority of peptides generated presented low MWs, with fractions eluting below 14 kDa, particularly after 4 h of hydrolysis. This prevalence of small peptides is consistent with the absence of plasma proteins in the CF fraction, which in WBH contributes with larger MW components such as fibrinogen (~340 kDa) [35] and globulins (α-globulins ~93 kDa). Thus, compared to WBH, CFH displayed a higher proportion of small peptides, reflecting both its distinct protein composition (dominated by hemoglobin and membrane-associated proteins) and the efficiency of alcalase in breaking them into short fragments.

Taking into account the comparable hydrolytic efficiency between the 5% and 2.5% enzyme treatments, along with considerations of process cost-effectiveness, the condition of 2.5% (E/S) for 4 h was selected for the scale-up of CF hydrolysis. This condition was deemed optimal for achieving significant peptide release while minimizing enzyme usage.

### 3.2. Characterization of Scaled-Up Porcine Blood Hydrolysates

Following the optimization of hydrolysis conditions, scaled-up batches of both hydrolysates (WBH and CFH) were produced, freeze-dried and subjected to compositional analysis.

#### 3.2.1. Degree of Hydrolysis, Total Protein and Free Amino Acid Content

WBH exhibited a significantly higher DH (59.5 ± 2.6%) compared to CFH (30.8 ± 3.3%), suggesting that proteins present in whole blood are more readily accessible to enzymatic cleavage than those in the cellular fraction. As commonly reported for the TNBS method, the absolute DH values obtained may be higher than those determined by alternative approaches such as OPA or pH-stat. In the present study, DH was used as a relative parameter to compare enzymatic susceptibility between substrates and processing conditions.

This observation in DH between WBH and CFH is consistent with the predominance of hemoglobin and membrane-associated proteins in the red cell fraction, which may display lower susceptibility to enzymatic hydrolysis due to their structural complexity and strong interactions with cell membranes. Recent research has shown that hemoglobin can redistribute and bind to red blood cell membranes, forming stable complexes that reduce cellular deformability and likely hinder protease access and enzymatic action [36].

Despite the variation in DH, both WBH and CFH exhibited high protein contents, reaching 90.3 ± 1.4% and 85.5 ± 2.0%, respectively, confirming that porcine blood hydrolysates are highly protein-dense ingredients with minimal non-protein fractions. This is in line with previous reports on blood-derived products such as the commercial porcine plasma hydrolysate PEPTEIVA^®^, which contains approximately 76% protein, of which more than 85% corresponds to peptides smaller than 10 kDa [37].

To further characterize these hydrolysates, the free amino acid compositions of WBF and CFH are detailed in Table 1. CFH presented a higher total content of free amino acids (45.20 mg/g protein) compared to WBH (30.24 mg/g protein), suggesting that the hydrolysis of the cellular fraction released a greater pool of small nitrogenous compounds despite its lower overall DH.

In CFH, glutamic acid (9.02 mg/g protein), aspartic acid (7.73 mg/g protein) and alanine (8.82 mg/g protein) were particularly abundant. These amino acids play important roles in human health: glutamic acid functions as a key precursor of the neurotransmitter γ-aminobutyric acid (GABA) and contributes to nitrogen and energy metabolism [38]; aspartic acid is involved in hormone regulation and plays a role in the development, metabolism and function of the nervous system [39] and alanine is central to glucose–alanine cycling, facilitating gluconeogenesis and helping to regulate blood sugar levels during fasting or physical stress [40].

WBH was enriched in branched-chain amino acids (BCAAs), namely leucine (8.94 mg/g protein), isoleucine (3.53 mg/g protein) and valine (3.02 mg/g protein). These essential amino acids are mainly catabolized in muscle rather than the liver and play key roles in protein turnover and metabolic regulation. In particular, leucine stimulates protein synthesis through activation of the mTORC1 pathway, a mechanism strongly associated with muscle growth and recovery [41].

These results reinforce the potential of porcine blood fractions to be efficiently valorized into protein-rich hydrolysates with complementary characteristics, suitable for targeted applications in functional foods or nutraceuticals.

#### 3.2.2. Bioactive Properties of Porcine Blood Hydrolysates

The assessment of bioactive properties in protein hydrolysates provides important insights into their potential health-promoting effects. In this study, the antioxidant and antihypertensive activities of porcine blood hydrolysates were evaluated both before and after simulated gastrointestinal digestion in order to determine their stability and potential physiological relevance (Table 2). Previous research has already demonstrated that animal blood can serve as a source of bioactive peptides, principally with antioxidant potential [13,17,42,43]. However, the changes in bioactivity following gastrointestinal digestion have not been fully clarified. Therefore, evaluating the activity of porcine blood hydrolysates under simulated digestive conditions is crucial to better understand their functional value in vivo.

##### Antioxidant Capacity and Impact of Simulated Gastrointestinal Digestion

Antioxidant activity is a broad term that refers to the capacity of compounds to inhibit or neutralize the detrimental effects of free radicals or reactive oxygen species (ROS), thereby protecting cells and tissues from oxidative damage. It represents a key functional property of protein hydrolysates and is largely influenced by amino acid composition, peptide size and peptide sequence [44]. In the present study, the antioxidant potential of WBH and CFH was evaluated using the ABTS and ORAC assays (Table 2).

When assessed by the ABTS assay, CFH exhibited significantly higher antioxidant activity compared to WBH (*p* < 0.05). This enhanced performance may be attributed to the higher levels of free amino acids and low MW peptides (<14 kDa) present in CFH. Small peptides have been associated with enhanced antioxidant capacity, primarily due to their greater reactivity and ability to interact effectively with free radicals [45]. Despite the higher DH observed in WBH, this factor did not translate into improved ABTS radical scavenging. In agreement with these findings, Boonkong et al. [46] reported that the DH of bovine blood hydrolysates was not directly correlated with ABTS radical scavenging activity. This suggests that other compositional features, such as polarity, amino acid sequence and hydrophobicity, play a decisive role. Moreover, a higher abundance of specific residues, including Gly, Ala, Leu, Trp, Tyr, Met, Arg, Phe, and Val, has been associated with enhanced radical scavenging capacity [17].

The results of the ORAC assay revealed no significant differences between WBH and CFH (*p* > 0.05), suggesting that both hydrolysates have a comparable capacity to scavenge peroxyl radicals under conditions simulating physiological oxidative stress.

Previous studies have also reported significant antioxidant activity in animal by-products, particularly in porcine, bovine and chicken blood hydrolysates [13,17,42,43].

The divergent outcomes between ABTS and ORAC assays underscore the importance of employing multiple complementary methods to comprehensively evaluate antioxidant activity. Whereas ABTS is particularly sensitive to hydrophilic compounds and small nitrogenous molecules, ORAC provides a more biologically relevant assessment of peroxyl radical neutralization [47].

To further evaluate the stability and potential bioactivity of the hydrolysates under physiological conditions, the antioxidant activity of WBH and CFH was also assessed after simulated gastrointestinal digestion (Table 2). In vitro digestion significantly affected the antioxidant activity of the hydrolysates, but the impact varied depending on the assay employed.

In the ABTS assay, antioxidant activity was maintained after digestion, with no significant reduction compared to the undigested hydrolysates (*p* > 0.05). In contrast, the ORAC assay revealed a decrease in antioxidant activity following digestion for both WBH and CFH (*p* < 0.05). This suggests that peroxyl radical-scavenging capacity is particularly sensitive to gastrointestinal conditions. The loss of activity may be explained by the extensive proteolysis that disrupts bioactive peptide sequences, generating fragments with reduced capacity to neutralize peroxyl radicals.

In this context, the application of food matrix interactions and encapsulation strategies may represent effective approaches to enhance the gastrointestinal stability of porcine blood hydrolysates, thereby protecting their bioactive peptides from hydrolytic degradation and enhancing their potential efficacy in vivo [48,49].

##### ACE Inhibitory Potential and Impact of Simulated Gastrointestinal Digestion

Antihypertensive peptides derived from food proteins have gained considerable interest as safer alternatives to conventional drugs. The regulation of elevated blood pressure is primarily governed by the renin–angiotensin–aldosterone system (RAAS), in which ACE plays a central role in maintaining the renin–angiotensin balance. ACE is a zinc-dependent metallopeptidase that catalyzes the conversion of angiotensin I into the potent vasoconstrictor angiotensin II, thereby promoting aldosterone secretion, vascular constriction and ultimately increased blood pressure [50]. Therefore, compounds capable of inhibiting ACE activity represent promising agents for the prevention and management of hypertension. In the present study, the anti-hypertensive potential of WBH and CFH was evaluated using the ACE inhibition assay (Table 2).

CFH exhibited a significantly lower IC_50_ value (59.5 ± 3.2 µg protein/mL) compared to WBH (153.2 ± 10.7 µg protein/mL), reflecting a stronger ACE inhibitory potential. This enhanced bioactivity of CFH may be attributed to its higher content of small peptides (<14 kDa) and free amino acids, which are more likely to generate ACE-inhibitory effects. In fact, ACE inhibition is generally associated with short-chain peptides composed of 2–12 amino acids [51]. Porcine hemoglobin hydrolysates obtained with pepsin, trypsin and papain have been reported to display relatively weak ACE inhibitory activity, with IC_50_ values of 1190.0, 8790.0 and 2210.0 µg/mL, respectively [52]. The porcine blood hydrolysates evaluated in this study demonstrate excellent antihypertensive potential, as their IC_50_ values (59.5–153.2 µg protein/mL) are considerably lower than the reference threshold of 500 µg protein/mL [13].

After simulated gastrointestinal digestion, WBH maintained its ACE inhibitory capacity, whereas CFH showed a decrease, resulting in comparable IC_50_ values (135.1 ± 13.2 µg protein/mL for WBH and 118.3 ± 12.2 µg protein/mL for CFH; *p* > 0.05). These results suggest that gastrointestinal proteolysis alters peptide profiles, as previously demonstrated for the peptide IIVFGRQLL, whose ACE inhibitory activity decreased by 39.0% after digestion due to pepsin-mediated cleavage into smaller fragments and free amino acids, thereby reducing the concentration of the original active sequence [53].

Taken together, the results indicate that porcine blood hydrolysates possess relevant antioxidant and antihypertensive properties, supporting their potential as a source of bioactive peptides with possible applications in health-promoting foods.

#### 3.2.3. Techno-Functional Properties

In addition to their well-documented bioactive properties, protein hydrolysates may also contribute to the techno-functional quality of foods. Specific peptide fractions have been reported to improve functionalities such as emulsifying and stabilizing capacity, water- and oil-holding ability, and even taste modulation, thereby broadening their potential applications in food formulation [54].

Emulsifying capacity refers to the ability of proteins to adsorb at the oil-water interface, reducing interfacial tension and facilitating the formation of stable droplets. It is commonly expressed as the amount of oil that can be emulsified per gram of protein under standardized conditions [14].

WBH and CFH exhibited similar emulsifying properties, with approximately 40% (*p* > 0.05). This capability was stable to heat treatment, also demonstrating a good emulsifying stability (~40%) (Table 3). Protein hydrolysates can stabilize oil-in-water emulsions through the exposure of hydrophilic and hydrophobic amino acid residues during enzymatic hydrolysis. Hydrophobic peptides are readily adsorbed at the interfacial layer, while hydrophilic peptides remain in the aqueous phase; together, these residues act as surfactants that promote emulsion stability [18]. The emulsifying levels of WBH and CFH were lower than those reported for fibrinogen (~100%), γ-globulins (70–85%) and whole plasma (>80%) [14], fractions known for their strong interfacial activity. Nevertheless, when compared with other blood proteins, their performance was superior to porcine albumin (25–30%) and decolored globin (<15%), and similar to hemoglobin at moderate concentrations (40–50% at >6 g/L)) [14].

The oil absorption capacity is an important techno-functional property, as it directly affects the sensory attributes of food products. The mechanism of oil absorption is mainly associated with the physical entrapment of oil within the protein matrix, which is influenced by the bulk density of the protein and its structural characteristics [55].

WBH and CFH both exhibited oil absorption capacity, with CFH showing values nearly four times higher than WBH (4.79 vs. 1.31 g oil/g dry extract; Table 3). This difference can be attributed to the distinct protein composition of the two substrates. CFH, derived from the cellular fraction, is enriched in hemoglobin and membrane-associated proteins, which expose more hydrophobic residues and thereby enhance oil-binding capacity. Conversely, WBH includes plasma proteins such as albumin, fibrinogen and globulins, which are predominantly hydrophilic and less effective in oil entrapment. The higher oil absorption observed in CFH can be explained by the established relationship between oil-binding potential and protein surface hydrophobicity [56]. Moreover, bulk density and peptide conformation after enzymatic hydrolysis may also contribute to these differences [55].

## 4. Conclusions

Porcine blood hydrolysates produced with alcalase proved to be a valuable source of multifunctional ingredients, combining antioxidant and antihypertensive (ACE-inhibitory) bioactivities with techno-functional properties such as emulsification and oil absorption. WBH showed higher hydrolysis, whereas CFH exhibited stronger ABTS activity, more pronounced ACE inhibition and greater oil absorption capacity, highlighting distinct functional advantages that may be exploited in targeted applications within functional foods and nutraceuticals. Beyond their health-promoting potential, this work emphasizes porcine blood, a major slaughterhouse by-product, as a promising raw material for sustainable valorization within a circular bioeconomy framework. A key strength of this study lies in the integrated evaluation of bioactive, techno-functional and digestive stability properties, combined with a side-by-side comparison of WB and its CF under identical hydrolysis conditions. The use of non-anticoagulated blood and the inclusion of a scale-up step further enhance the practical relevance and industrial applicability of the findings. Based on existing evidence, the use of porcine blood–derived hydrolysates in food systems does not present specific contraindications, provided that their production complies with established regulatory standards for edible animal-derived ingredients.

Future research should prioritize the identification of bioactive peptide sequences, in vivo validation of their health effects and strategies such as encapsulation to enhance gastrointestinal stability. A more detailed kinetic analysis at shorter reaction times may be addressed in future studies to further elucidate early-stage hydrolysis mechanisms. From an industrial perspective, optimizing large-scale processing, ensuring regulatory compliance and evaluating consumer acceptance will be critical to transition porcine blood hydrolysates from experimental products to high-value functional ingredients.

## Figures and Tables

**Table 1:** Composition of free amino acids of porcine blood hydrolysates.

mg/g Protein	WBH	CFH
Aspartic acid	0.80 ± 0.05	7.73 ± 0.32
Glutamic acid	1.91 ± 0.06	9.02 ± 0.24
Cysteine	0.07 ± 0.00	0.06 ± 0.01
Asparagine	0.06 ± 0.00	n.d.
Serine	0.80 ± 0.05	1.07 ± 0.01
Histidine	1.38 ± 0.06	n.d.
Glutamine	0.48 ± 0.05	0.17 ± 0.05
Threonine	1.19 ± 0.06	1.12 ± 0.00
Arginine	0.68 ± 0.02	1.30 ± 0.00
Alanine	2.78 ± 0.06	8.82 ± 0.15
Tyrosine	1.91 ± 0.06	1.07 ± 0.03
Valine	3.02 ± 0.20	6.06 ± 0.06
Methionine	1.28 ± 0.10	0.66 ± 0.02
Tryptophan	0.87 ± 0.01	0.69 ± 0.01
Phenylalanine	0.55 ± 0.00	1.43 ± 0.04
Isoleucine	3.53 ± 0.10	0.62 ± 0.02
Leucine	8.94 ± 0.20	5.37 ± 0.05
Total	30.24	45.20

n.d. not detected.

**Table 2:** Antioxidant capacity (ABTS and ORAC) and ACE-inhibitory potential of porcine blood hydrolysates before and after simulated gastrointestinal tract (GIT).

Bioactive Property	Condition	WBH	CFH
Antioxidant capacity			
ABTS (mg AAE/g)	Before GIT	11.1 ± 0.2 ^a^	14.1 ± 0.1 ^b^
	After GIT	11.5 ± 0.7 ^a^	13.9 ± 0.2 ^b^
ORAC (mg TE/g)	Before GIT	180.2 ± 4.9 ^d^	166.8 ± 15.5 ^d^
	After GIT	107.3 ± 12.8 ^e^	102.0 ± 23.0 ^e^
ACE inhibitory potential			
IC_50_ (µg of protein/mL)	Before GIT	153.2 ± 10.7 ^f^	59.5 ± 3.2 ^g^
	After GIT	135.1 ± 13.2 ^f^	118.3 ± 12.2 ^f^

Means with the same superscript are not statistically different (*p* ≥ 0.05).

**Table 3:** Techno-functional properties of porcine blood hydrolysates, including emulsifying activity, emulsifying stability and oil absorption capacity.

Techno-Functional Property	WBH	CFH
Emulsifying property (%)	44.3 ± 1.0 ^a^	42.1 ± 0.1 ^a^
Emulsifying stability (%)	44.0 ± 0.4 ^a^	43.6 ± 0.5 ^a^
Oil absorption capacity (g oil/g dry extract)	1.31 ± 0.07 ^b^	4.79 ± 0.15 ^c^

Means with the same superscript are not statistically different (*p* ≥ 0.05).

## Data Availability

The original contributions presented in this study are included in the article. Further inquiries can be directed to the corresponding author.

## Abbreviations

AAE	Ascorbic acid equivalents
AAPH	2,2′-Azobis(2-amidinopropane) dihydrochloride
ABTS	2,2′-Azino-bis(3-ethylbenzothiazoline-6-sulfonic acid)
ACE	Angiotensin-converting enzyme
ANOVA	Analysis of variance
AUC	Area under the curve
BCAAs	Branched-chain amino acids
CF	Red cell fraction
CFH	Red cell fraction hydrolysate
DH	Degree of hydrolysis
E/S	Enzyme-to-substrate ratio
FPLC	Fast protein liquid chromatography
GIT	Gastrointestinal tract
HPLC	High-performance liquid chromatography
IC_50_	Concentration required to inhibit 50% of enzymatic activity
LSD	Least significant difference
MW	Molecular weight
n.d.	Not detected
OPA	Orthophthalaldehyde
ORAC	Oxygen radical absorbance capacity
ROS	Reactive oxygen species
RP-HPLC	Reverse-phase high-performance liquid chromatography
SPSS	Statistical Package for the Social Sciences
TE	Trolox equivalents
TNBS	2,4,6-Trinitrobenzenesulfonic acid
WB	Whole blood
WBH	Whole blood hydrolysate
